# Sinomenine Hydrochloride Inhibits Human Glioblastoma Cell Growth through Reactive Oxygen Species Generation and Autophagy-Lysosome Pathway Activation: An In Vitro and In Vivo Study

**DOI:** 10.3390/ijms18091945

**Published:** 2017-09-11

**Authors:** Yumao Jiang, Yue Jiao, Zhiguo Wang, Tao Li, Yang Liu, Yujuan Li, Xiaoliang Zhao, Danqiao Wang

**Affiliations:** Beijing Key Laboratory of Traditional Chinese Medicine Basic Research on Prevention and Treatment of Major Diseases, Experimental Research Center, China Academy of Chinese Medical Sciences, Beijing 100000, China; 13683301305@163.com (Y.J.); Jiaoyue_medicine@163.com (Y.J.); zhgw68_8@tom.com (Z.W.); hndxlitao@163.com (T.L.); echoinapril@163.com (Y.L.); lyjdcl@163.com (Y.L.); Zhaoxiaoliang1218@aliyun.com (X.Z.)

**Keywords:** glioblastoma, sinomenine hydrochloride, autophagy, reactive oxygen species, lysosome

## Abstract

Glioblastoma is the most common malignant primary brain tumor, and it is one of the causes of cancer fatality in both adult and pediatric populations. Patients with glioblastoma require chemotherapy after surgical resection and radiotherapy. Therefore, chemotherapy constitutes a viable approach for the eradication of glioblastoma cells. In this study, the anti-tumor activity of sinomenine hydrochloride (SH) was evaluated in U87 and SF767 cells in vitro and in vivo. The results showed that SH potently inhibited U87 and SF767 cell viability and did not cause caspase-dependent cell death, as demonstrated by the absence of significant early apoptosis and caspase-3 cleavage. Instead, SH activated an autophagy-mediated cell death pathway, as indicated by the accumulated microtubule-associated protein light chain 3B (LC3B)-II, triggered autophagic flux and enhanced cell viability after pretreatment with autophagy inhibitors. SH-mediated autophagy in the two cell lines was implicated in reactive oxygen species (ROS) generation, protein kinase B (Akt)-mammalian target of rapamycin (mTOR) pathway suppression and c-Jun NH2-terminal kinase (JNK) pathway activation. The ROS antioxidant *N*-acetylcysteine (NAC), the Akt-specific activator insulin-like growth factor-1 (IGF-1) and the JNK-specific inhibitor SP600125 attenuated SH-induced autophagy. Moreover, ROS activated autophagy via the Akt-mTOR and JNK pathways. Additionally, SH treatment may promote lysosome biogenesis through activating transcription factor EB (TFEB). The in vivo study found that SH effectively suppressed glioblastoma growth without exhibiting significant toxicity. In conclusion, our findings reveal a novel mechanism of action of SH in cancer cells via the induction of autophagy through ROS generation and autophagy-lysosome pathway activation; these findings also supply a new potential therapeutic agent for the treatment of human glioblastoma.

## 1. Introduction

Glioblastoma is one of the most aggressive and malignant brain tumors in the central nervous system, and conventional cancer therapies have failed to exert positive effects on human glioblastoma [1,2]. In general, patients with glioblastoma have a poor prognosis, which may be partially attributed to the inherent apoptosis-resistant phenotype of the malignancy. The development of new therapies is urgently required for the treatment of glioblastoma.

The traditional Chinese medicinal plant *Sinomenium acutum* Rehd. et Wils. (Fam. Menispermaceae) has been employed to effectively remedy rheumatoid arthritis for centuries [3]. Sinomenine (7,8-didehydro-4-hydroxy-3,7-dimethoxy-17-methylmorphinan-6-one, C19H23NO4), is the major effective alkaloid derived from the plant. SH is a hydrochloride form of sinomenine that is widely applied in the clinical treatment of rheumatoid diseases due to its anti-immune and anti-inflammatory effects [4]. Recently, Zhu et al. identified the mechanisms of the effect of sinomenine on decreasing analgesic tolerance [5,6]. In addition, the anti-tumor activity of sinomenine has received increasing attention, and several studies have examined the anti-tumor activity of SH or sinomenine in hepatic cancer, mammary tumors and colon carcinoma [7,8,9]. However, the underlying mechanisms of the anti-cancer effects of SH remain unclear. Many studies have found that sinomenine shows positive activity in diseases of the central nervous system, such as neurodegenerative disorders, ischemia/reperfusion brain damage and experimental autoimmune encephalomyelitis [10,11,12,13,14], and our previous studies have shown that the prescription of CQM, whose main active ingredient is SH, has a significant analgesic effect on neurogenic pain and cancerous pain [15,16]. Therefore, we sought to determine whether SH could be used to treat human glioblastoma of the central nervous system. Our results indicate that SH suppresses U87 and SF767 cell proliferation through an autophagy mechanism. Targeting autophagy regulators to trigger autophagy has been reported to be an attractive therapeutic strategy for cancer [17]. To our knowledge, there has been no previous study on SH-induced autophagy reported in the literature. 

Autophagy is regarded as a critical adaptive and homeostatic process delivering organelles and cytoplasmic proteins to lysosomes for digestion. Dysregulation of autophagy is related to the suppression of tumorigenesis and malignant transformation [18,19]; however, its role in glioblastoma is still unclear. The currently available studies on autophagy in different cell types under various cellular conditions show conflicting evidence regarding its role in cellular death [20,21]. Although the biological functions of autophagy may be context- dependent, there are many reports showing that some natural compounds or therapeutic drugs can induce caspase-independent autophagic cell death by activating autophagy signaling pathways [22,23].

ROS play an important role in cancer cell death. The level of intracellular ROS is increased under stress conditions, and ROS could act as signaling molecules inducing caspase-independent autophagy-mediated cancer cell death [24,25]. Moreover, recent reports have indicated that the Akt-mTOR pathway inhibition has been confirmed to activate autophagy and suppress cancer cell growth [26,27], and the suppression of mTOR activity by rapamycin (Rapa) can induce autophagy and restrain cancer cell growth [28]. Phosphorylation of JNK is another crucial factor in autophagy-mediated cell death in different cancer cells [29,30,31]. In addition to the two signaling pathways mentioned above, the lysosome has a key function in the process of autophagic flux. Previous research has demonstrated that the major signaling molecule regulating lysosomal biogenesis is TFEB, a basic helix-loop-helix leucine zipper transcription factor [32], and mTOR inhibitors, such as PP242 and torin1, are currently primarily regarded as activators of TFEB, by means of triggering its nuclear translocation [33,34]. Based on these observations, we hypothesized that SH suppresses human glioblastoma cell growth by regulating these signaling pathways.

As a step toward applying SH as a chemotherapeutic agent for treating human glioblastoma, we characterized the molecular mechanisms by which SH inhibits the proliferation of U87 and SF767 cells in the present study. Our results reveal an innovative mechanism of action of SH in triggering autophagy, but not apoptosis, in both human glioblastoma cells through ROS generation and autophagy-lysosome pathway activation.

## 2. Results

### 2.1. SH Inhibits Human Glioblastoma Cell Proliferation through a Caspase-Independent Pathway

**Figure 1:**
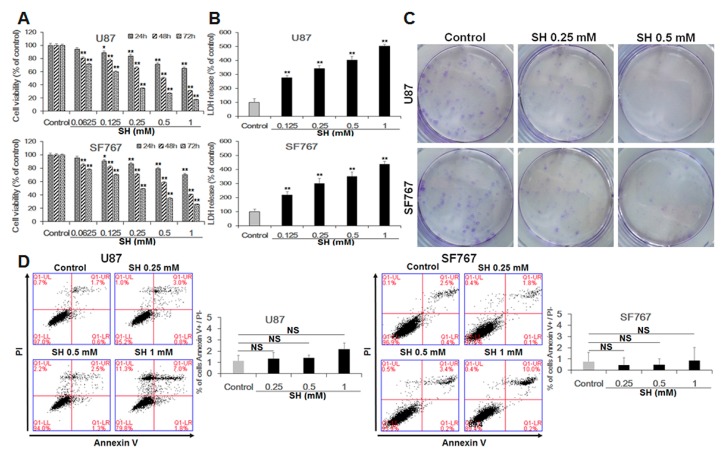

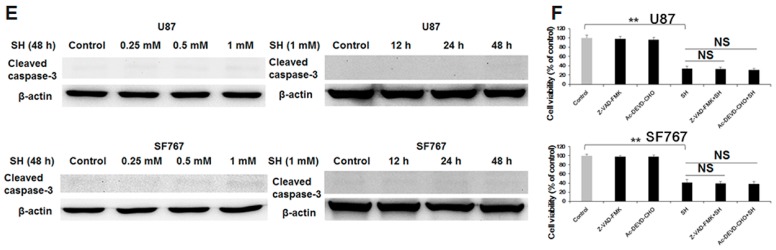
SH inhibits cell proliferation through a caspase-independent pathway in U87 and SF767 cells. (**A**) Dose- and time-dependent effects of SH on cell viability. U87 and SF767 cells were treated with different concentrations of SH (0.0625, 0.125, 0.25, 0.5, 1 mM) for 24, 48 or 72 h, and cell viability was assessed via cell counting kit-8 (CCK-8) assays; (**B**) The cells were treated with 0.125, 0.25, 0.5, or 1 mM SH for 48 h, and LDH assays were performed; (**C**) U87 and SF767 cells viability upon the indicated concentrations of SH treatment was measured via colony formation assays; (**D**) Dose-dependent effects of SH on apoptosis. After treatment with the indicated concentrations of SH for 48 h, apoptosis was detected through Annexin V-FITC/PI staining and quantitative analysis of the percentage of Annexin V-positive and PI-negative cells compared with the control groups; (**E**) Extent of apoptosis, as observed by the levels of cleaved caspase-3 in U87 and SF767 cells, in the two cell lines treated with the indicated concentrations of SH for 48 h or at 1 mM for the indicated times; (**F**) Effects of the caspase-3 inhibitor Ac-DEVD-CHO and the pancaspase inhibitor Z-VAD-FMK on human glioblastoma cell death induced by SH. U87 and SF767 cells were preincubated with Z-VAD-FMK (50 µM) and Ac-DEVD-CHO (50 µM) for 1 h, followed by co-incubation with SH (1 mM) for 48 h. Each image is representative of *n* = 3 experiments. The results shown in E are one representative Western blot, *n* = 3, and β-actin served as the loading control. All data are shown as the mean ± SEM, *n* = 3. * *p* < 0.05, ** *p* < 0.01, versus the control. NS, not significant versus the control or SH treatment alone.

SH is very effective in inhibiting cell proliferation in various types of human cancer cells. Previous studies have revealed that SH induces cell death via apoptosis in hepatoma and lung cancer cells [8,35]. We therefore examined whether SH could also cause cell death in U87 and SF767 cells via apoptosis. SH decreased cell viability in a dose- and time-dependent manner in U87 and SF767 cells (Figure 1A). LDH release assays demonstrated that SH increased cell death in a dose-dependent manner (Figure 1B). In addition, as shown in Figure 1C, we found that in the presence of 0.5 mM SH, little colonies were formed in the two cell lines, indicating that, compared with the reproductive potential of the control cells, the reproductive potential of the U87 and SF767 cells was inhibited by SH. To assess the early events of apoptosis, we treated U87 and SF767 cells with SH for 48 h. Then, phosphatidylserine levels at the cell surface were measured via Annexin V-fluorescent isothiocyanate (FITC) /PI staining. The percentage of Annexin V-FITC-positive/PI-negative cells in SH-treated U87 and SF767 cells was not significantly different from those of the control group (Figure 1D), demonstrating that SH induced no or little apoptosis in U87 and SF767 cells. This was confirmed by detecting the levels of cleaved caspase-3 (Figure 1E) after SH treatment in the two cell lines. To investigate whether cell death was caspase-independent, we further observed the effects of the pancaspase inhibitor Z-VAD-FMK and the caspase-3 inhibitor Ac-DEVD-CHO on SH-treated U87 and SF767 cells. The results suggested that the caspase inhibitors had no influence on cell death induced by SH (1.0 mM) (Figure 1F). Taken together, these data reveal that SH inhibits human glioblastoma cell growth through a caspase-independent pathway.

### 2.2. SH Inhibits Human Glioblastoma Cell Viability through Autophagy

Because SH-induced cell death in U87 and SF767 cells did not proceed via apoptosis, we examined whether SH-induced cell death is due to programmed cell death type II, autophagy. To determine whether SH induces autophagy-mediated cell death in U87 and SF767 cells, we performed the following experiments. 

**Figure 2:**
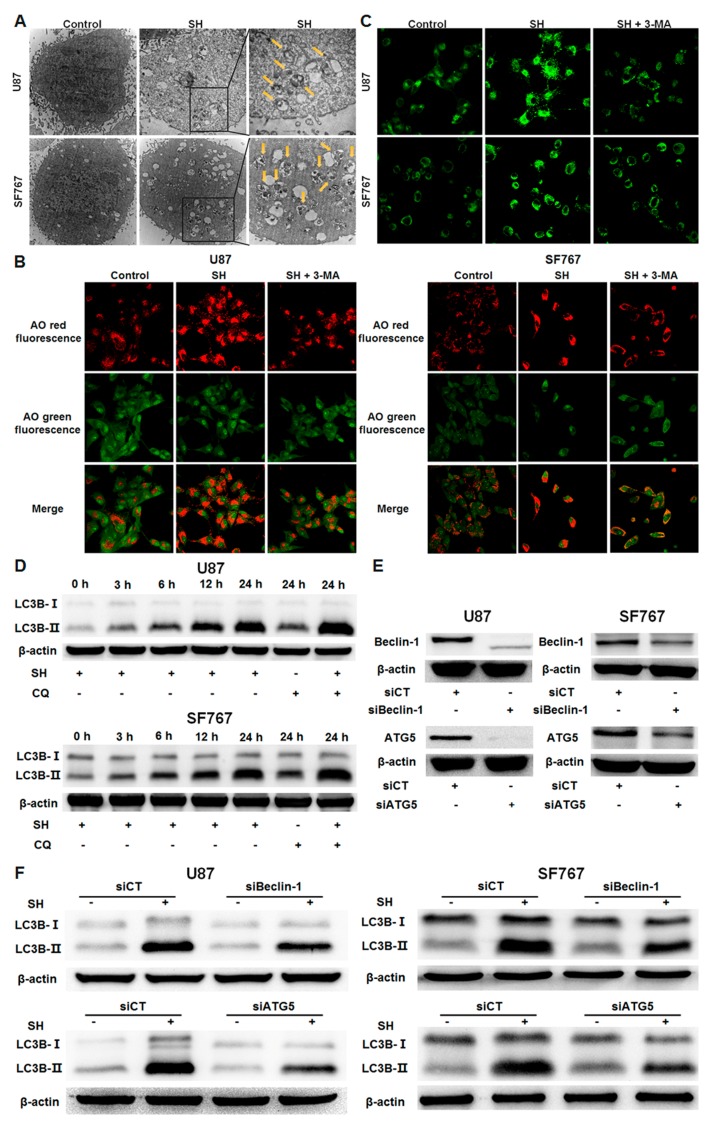

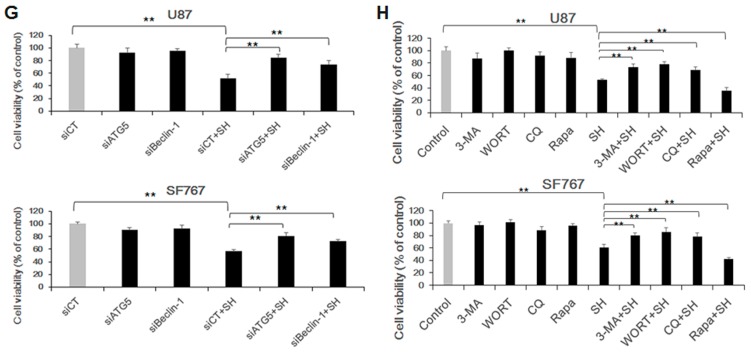
SH inhibits human glioblastoma cell viability through autophagy. (**A**) Representative transmission electron micrographs indicating the ultrastructure of U87 and SF767 cells. Various types of autophagic vesicles (arrowheads) are found within the cytoplasm in the two cell lines treated with SH (0.5 mM) for 24 h; (**B**) After 24 h of SH treatment (0.5 mM) in the presence or absence of 3-MA, the two cell lines were stained with AO and observed via confocal microscopy. AO produces red fluorescence in acidic autophagic vacuoles, and green fluorescence in cytoplasm and nucleus; (**C**) After 24 h of SH treatment (0.5 mM) in the presence or absence of 3-MA, the two cell lines were stained with MDC and imaged via confocal microscopy; (**D**) Effects of SH on LC3B-II levels in human glioblastoma cells. U87 and SF767 cells were treated with SH (0.5 mM) and/or CQ for the indicated times; (**E**) Efficiency of Beclin-1 and ATG5 knockdown. (**F**) Effects of ATG5 or Beclin-1 siRNA on SH-mediated autophagy. U87 and SF767 cells were transfected with a no-target control siRNA (siCT), ATG5 siRNA (siATG5), or Beclin-1 siRNA (siBeclin-1) for 24 h and then treated with or without SH (0.5 mM) for another 24 h; (**G**) Effects of ATG5 or Beclin-1 siRNA on SH-mediated inhibition of human glioblastoma cell proliferation; (**H**) Effects of autophagy inhibitors or activators on the SH-induced inhibition of human glioblastoma cell growth. Each image is representative of *n* = 3 experiments. The results shown in (**D**–**F**) are representative Western blots, *n* = 3, and β-actin served as the loading control. All data are shown as the mean ± SEM, *n* = 3. ** *p* < 0.01, versus the control or SH treatment alone.

First, autophagic activity was evaluated via the formation of autophagosomes using transmission electron microscopy, which is one of the most widely accepted methods for detecting autophagy [36]. When the SH-treated cells were observed under a transmission electron microscope, double- or multi-membrane structures containing high-electron-density substances characteristic of autolysosomes and autophagosomes were found (Figure 2A).

Second, acridine orange (AO) staining was used to monitor acidic vesicular organelles in U87 and SF767 cells. As shown in Figure 2B, SH treatment significantly increased the quantity of acidic vesicular organelles in U87 and SF767 cells, and the autophagy inhibitor 3-methyladenine (3-MA) partially blocked the increase in the number of acidic vesicular organelles induced by SH. In addition, we investigated the incorporation of monodancylcadaverin (MDC), which is an indicator of mature autophagic vacuoles (AVs), such as autophagolysosomes [37]. SH treatment substantially enhanced MDC-stained AVs, which appeared as distinct dot-like structures distributed within the cytoplasm or in perinuclear regions of U87 and SF767 cells (Figure 2C). Similarly, 3-MA partially blocked the increase in AVs induced by SH.

Third, we detected the expression of LC3B-II, which is a marker of autophagy [38]. Elevated time-dependent expression of LC3B-II was found in SH-treated U87 and SF767 cells. Since changes in the accumulation of LC3B-II could result from either degradation in lysosomes or autophagosome formation, it was imperative to verify whether the enhancement of LC3B-II levels induced by SH was caused by decreased autophagosome degradation or enhanced autophagosome formation. Hence, the two cell lines were pretreated with the lysosomal inhibitor chloroquine (CQ) for 1 h and were then co-incubated with SH (0.5 mM) for 24 h. The inhibitor gave rise to a further augmentation of LC3B-II levels, demonstrating that SH increases autophagic flux, rather than obstructing lysosomal degradation (Figure 2D).

Fourth, to further verify these findings, we blocked the initiation of autophagosome formation with autophagy-related 6 (Beclin-1) or autophagy-related 5 (ATG5) siRNA (Figure 2E). ATG5 and Beclin-1 have been previously reported to specifically initiate autophagosome formation during autophagy [39,40]. As expected, the effect of SH in inducing increased expression of LC3B-II was weakened in the cells transfected with Beclin-1 or ATG5 siRNA (Figure 2F).

Fifth, to confirm the relationship between autophagy and human glioblastoma cell death induced by SH, we treated U87 and SF767 cells with SH (0.5 mM) for 48 h, and the percentage of viability in the two cell lines was found to be enhanced in Beclin-1 or ATG5 knockdown cells (Figure 2G) as well as the cells pretreated with the autophagy inhibitor (CQ, 3-MA, wortmannin (WORT)). In contrast, pretreatment with an autophagy activator (Rapa) substantially decreased the cell viability of the two cell lines treated with SH (Figure 2H).

Collectively, these findings supply powerful evidence that autophagic flux is augmented in U87 and SF767 cells treated with SH and that SH-mediated human glioblastoma cell death in the two cell lines is dependent, at least in part, on autophagy triggering.

### 2.3. SH Induces Autophagy-Mediated Cell Growth Inhibition via Ros Generation

To clarify whether ROS generation is related to the SH-mediated induction of autophagy and inhibition of human glioblastoma cell growth, we performed the following experiments.

**Figure 3:**
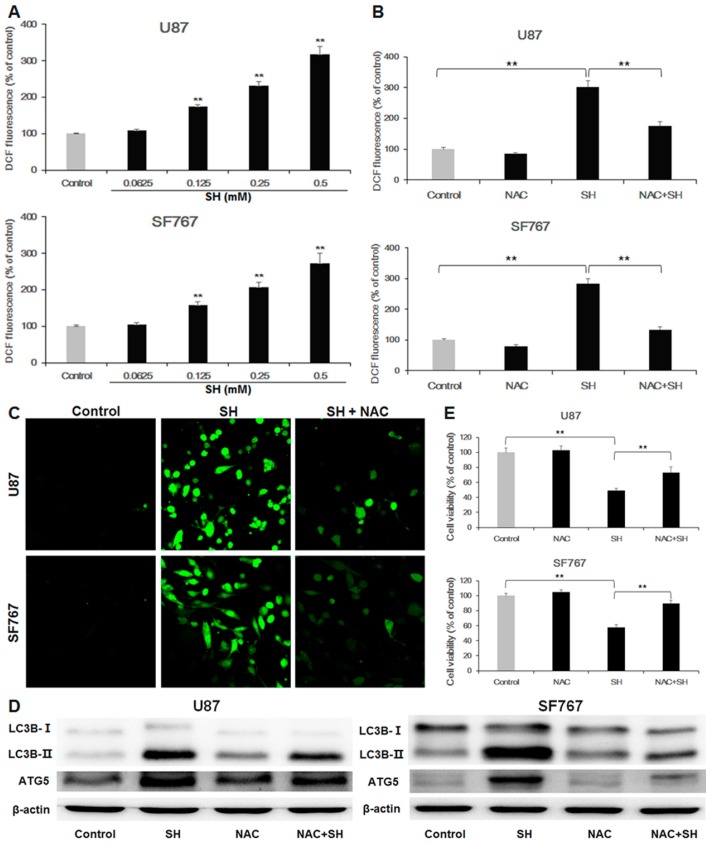
SH induces autophagy-mediated cell growth inhibition via ROS production (**A**) Dose-dependent effects of SH on intracellular ROS levels. U87 and SF767 cells were treated with different concentrations of SH for 24 h, and cellular ROS levels were detected using DCFH-DA probes, with the resulting fluorescence being read by a fluorescence microplate reader; (**B**) Effects of NAC on SH-mediated ROS generation. After pretreatment with the antioxidant NAC, followed by treatment with SH (0.5 mM) for 24 h, ROS levels were tested; (**C**) After pretreatment with the antioxidant NAC, followed by treatment with SH (0.5 mM) for 48 h, the ROS levels were imaged via confocal microscopy; (**D**) Effects of NAC on SH-mediated expression levels of LC3B-II and ATG5 in U87 and SF767 cells. (**E**) Effects of NAC on SH-mediated cell growth inhibition in U87 and SF767 cells. After pretreatment with the antioxidant NAC, followed by treatment with SH (0.5 mM) for 48 h, cell viability was measured via CCK-8 assays. Each image is representative of *n* = 3 experiments. The results shown in (**D**) are a representative Western blot, *n* = 3, and β-actin served as the loading control. All data are shown as the mean ± SEM, *n* = 3. ** *p* < 0.01, versus the control or SH treatment alone.

First, after treatment with SH, intracellular ROS levels were detected using 2′,7′-dichlorofluorescin diacetate (DCFH-DA) probes, quantified using a fluorescence microplate reader and imaged by a confocal microscopy. The results demonstrated that SH enhanced ROS generation in U87 and SF767 cells. In contrast, pretreatment with the ROS inhibitor NAC markedly attenuated the increase in the generation of ROS in the two cell lines treated with SH (Figure 3A–C).

Second, to verify the effect of ROS in SH-induced autophagy, we incubated SH with NAC. The results indicated that pretreatment with NAC could decrease LC3B-II accumulation and the expression levels of ATG5 in U87 and SF767 cells treated with SH (0.5 mM) for 24 h (Figure 3D).

Third, to further clarify the role of ROS on SH-mediated human glioblastoma cell death, we performed CCK-8 assays. The data suggested that pretreatment with NAC could increase cell viability in U87 and SF767 cells treated with SH (Figure 3E).

In summary, these observations revealed that SH treatment augments the levels of ROS in U87 and SF767 cells and that ROS generation induced by SH mediates autophagy triggering and the human glioblastoma cell death.

### 2.4. SH Induces Autophagy by Inhibiting the Akt-mTOR Pathway and Activating the JNK Pathway

**Figure 4:**
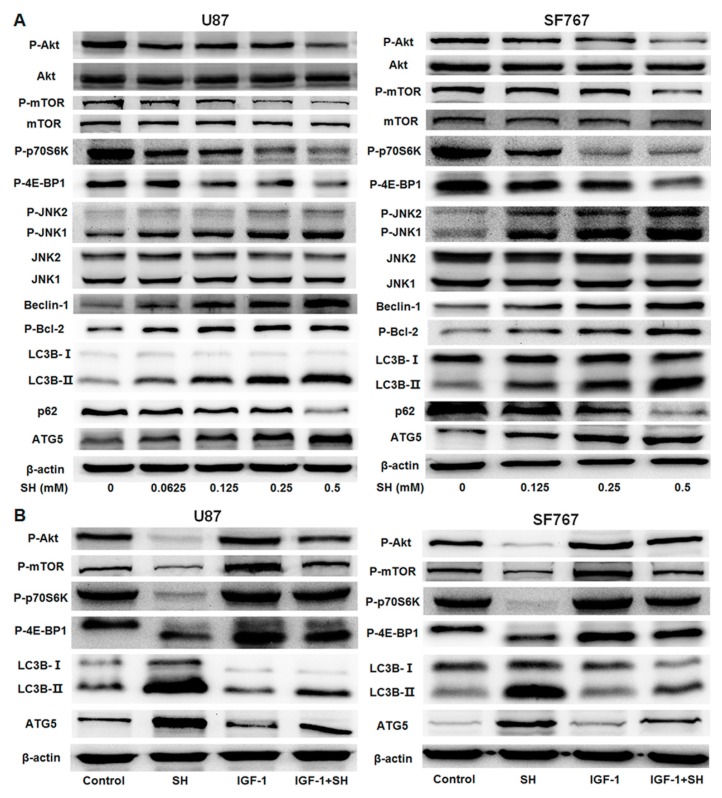

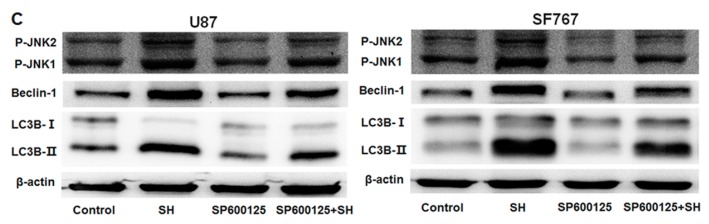
Inhibition of the Akt-mTOR pathway and activation of the JNK pathway are implicated in SH-mediated autophagy. (**A**) SH dose-dependently suppressed the Akt-mTOR pathway and activated the JNK pathway in U87 and SF767 cells, along with the induction of autophagy. The cells were treated with different concentrations of SH for 24 h; (**B**) Effects of IGF-1 on SH-mediated autophagy and suppression of the Akt-mTOR pathway in U87 and SF767 cells. The cells were treated with SH (0.5 mM) for 24 h in the absence or presence of IGF-1; (**C**) Effects of SP600125 on SH-mediated autophagy and the JNK pathway activation in U87 and SF767 cells. The cells were treated with SH (0.5 mM) for 24 h in the absence or presence of SP600125. β-actin served as the loading control. All blots shown are representative of *n* = 3 experiments, with similar results.

Western blot analysis was employed to evaluate whether the Akt-mTOR pathway is implicated in SH-mediated autophagy. The results showed that along with upregulation of LC3B-II and ATG5 levels and downregulation of sequestosome 1 (p62) acting as a cargo receptor for autophagic degradation of ubiquitinated targets, SH dose-dependently reduced the phosphorylation of mTOR, Akt, ribosomal protein S6 kinase (p70S6K) (an mTOR substrate) and eukaryotic translation initiation factor 4E-binding protein 1 (4E-BP1) (another downstream effector of mTOR) in U87 and SF767 cells (Figure 4A). In contrast, pretreatment with the Akt activator IGF-1 partially reversed the inhibition of the phosphorylation of Akt, mTOR, p70S6K, and 4E-BP1 and the enhanced expression levels of LC3B-II and ATG5 in the two cell lines treated with SH (Figure 4B). These observations reveal that inhibition of the Akt-mTOR pathway is related to SH-mediated autophagy in both human glioblastoma cell lines. 

Additionally, we investigated whether the JNK pathway is also involved in SH-mediated autophagy. As shown in Figure 4A, SH upregulated the expression levels of Beclin-1 and p-B-cell lymphoma-2 (Bcl-2) and enhanced the phosphorylated form of JNK1 in a dose-dependent manner in U87 and SF767 cells. In contrast, pretreatment with the JNK inhibitor SP600125 substantially attenuated Beclin-1 and LC3B-II levels and decreased the phosphorylation of JNK1 in the two cell lines treated with SH (Figure 4C). These results demonstrate that SH-induced autophagy also requires the JNK pathway activation in both human glioblastoma cell lines.

Collectively, these data indicate that SH-mediated autophagy is attributed to the activation of the JNK pathway and the inhibition of the Akt-mTOR pathway in both human glioblastoma cell lines.

### 2.5. ROS Generation Is Upstream of Akt-mTOR Pathway Inhibition and JNK Pathway Activation in SH-Mediated Autophagy

**Figure 5:**
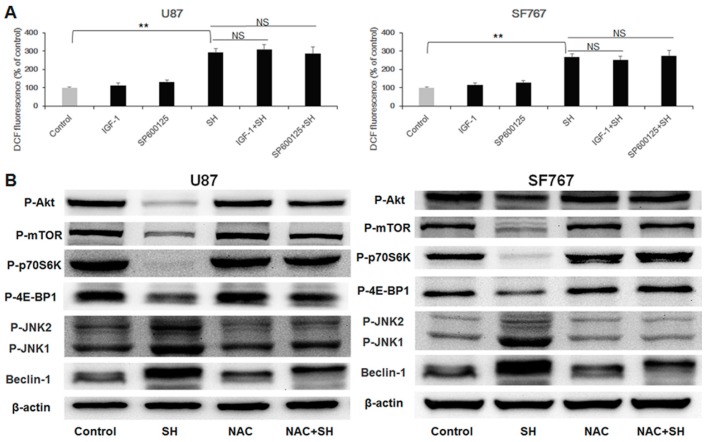
ROS production is upstream of JNK pathway activation and Akt-mTOR pathway inhibition in SH-mediated autophagy (**A**) Effects of IGF-1 and SP600125 on SH-induced ROS generation in U87 and SF767 cells. The cells were exposed to SH (0.5 mM) in the absence or presence of IGF-1 or SP600125 for 24 h, and intracellular ROS levels were investigated using DCFH-DA probes, with the resulting fluorescence being read by a fluorescence microplate reader; (**B**) Effects of NAC on SH-mediated Akt-mTOR pathway inhibition and JNK pathway activation in U87 and SF767 cells. The cells were treated with SH (0.5 mM) for 24 h in the absence or presence of NAC. β-actin was used as the loading control. All blots shown are representative of *n* = 3 experiments, with similar results. Data are expressed as the mean ± SEM, *n* = 3. ** *p* < 0.01, versus the control. NS, not significant versus the group treated with SH alone.

To further elucidate the relationship between ROS and the two mentioned signaling pathways, which are the Akt-mTOR and the JNK pathways in SH-induced autophagy, we assessed the effects of NAC, IGF-1, and SP600125. As shown in Figure 5A, the Akt activator IGF-1 and the JNK inhibitor SP600125 had no effect on SH-mediated ROS production. However, the antioxidant NAC eliminated the SH-mediated decrease in the phosphorylation of Akt, mTOR, 4E-BP1 and p70S6K as well as the increase in Beclin-1 levels and JNK1 phosphorylation (Figure 5B). In summary, the results revealed that ROS generation is the upstream process resulting in JNK pathway activation and Akt-mTOR pathway inhibition in U87 and SF767 cells treated with SH.

### 2.6. Effect of SH on Lysosomal Liogenesis

**Figure 6:**
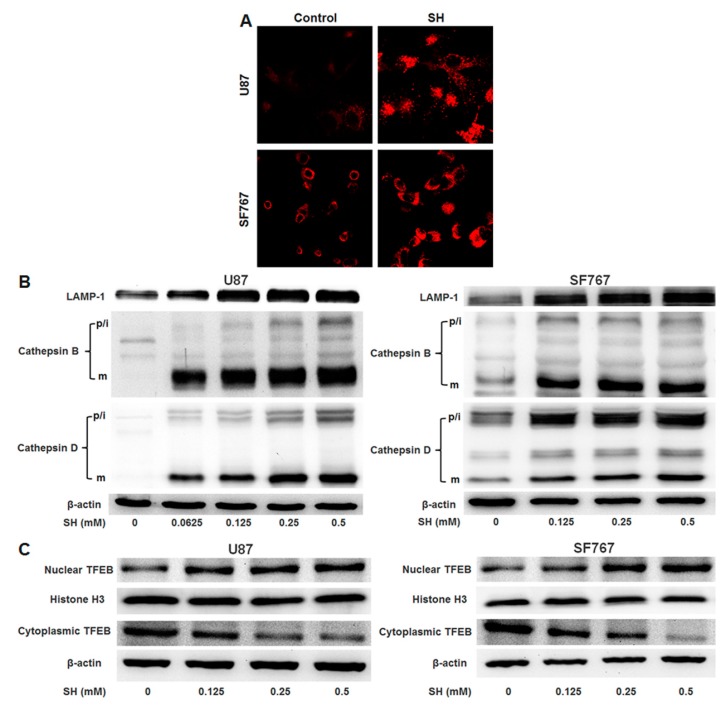
SH facilitates lysosomal biogenesis in U87 and SF767 cells. (**A**) After 24 h of SH (0.5 mM) treatment, the cells were stained with LTR and imaged via confocal microscopy; (**B**) U87 and SF767 cells were treated with the indicated concentrations of SH for 24 h. The expression levels of lysosome markers (LAMP1, cathepsin B, cathepsin D) were observed via Western blotting analysis. p/i, precursor/intermediate; m, mature form of cathepsin B and cathepsin D; (**C**) Effects of SH on the nuclear translocation of TFEB. U87 and SF767 cells were treated with the indicated concentrations of SH for 24 h. The expression levels of TFEB in the cytosolic and nuclear fractions were detected through Western blotting analysis. Histone H3 and β-actin were used as loading controls for the nuclear and cytoplasmic fractions, respectively. Each image is representative of *n* = 3 experiments. All blots shown are representative of *n* = 3 experiments, with similar results.

To evaluate the effect of SH on lysosomal biogenesis, we employed Lyso-Tracker Red (LTR) to detect lysosome levels in response to SH treatment. The results showed that, compared with the control groups, U87 and SF767 cells showed strongly enhanced the red fluorescence signal after SH treatment. Thus, SH increased the number of lysosomes in the two cell lines (Figure 6A). However, since the intensity of the Lyso-Tracker dye can be altered via a change in pH, we monitored the expression levels of lysosomal-associated membrane protein-1 (LAMP-1), a marker of lysosomal membranes. As illustrated in Figure 6B, we found that SH treatment for 24 h augmented the expression levels of LAMP-1 in the two cell lines in a dose-dependent manner; additionally, SH dose-dependently upregulated the expression of two major lysosomal cathepsins, cathepsin B and cathepsin D. To extend our findings, we confirmed that SH induced TFEB nuclear translocation in both human glioblastoma cell lines in a dose-dependent manner (Figure 6C).

### 2.7. SH inhibits Tumor Growth In Vivo

**Figure 7:**
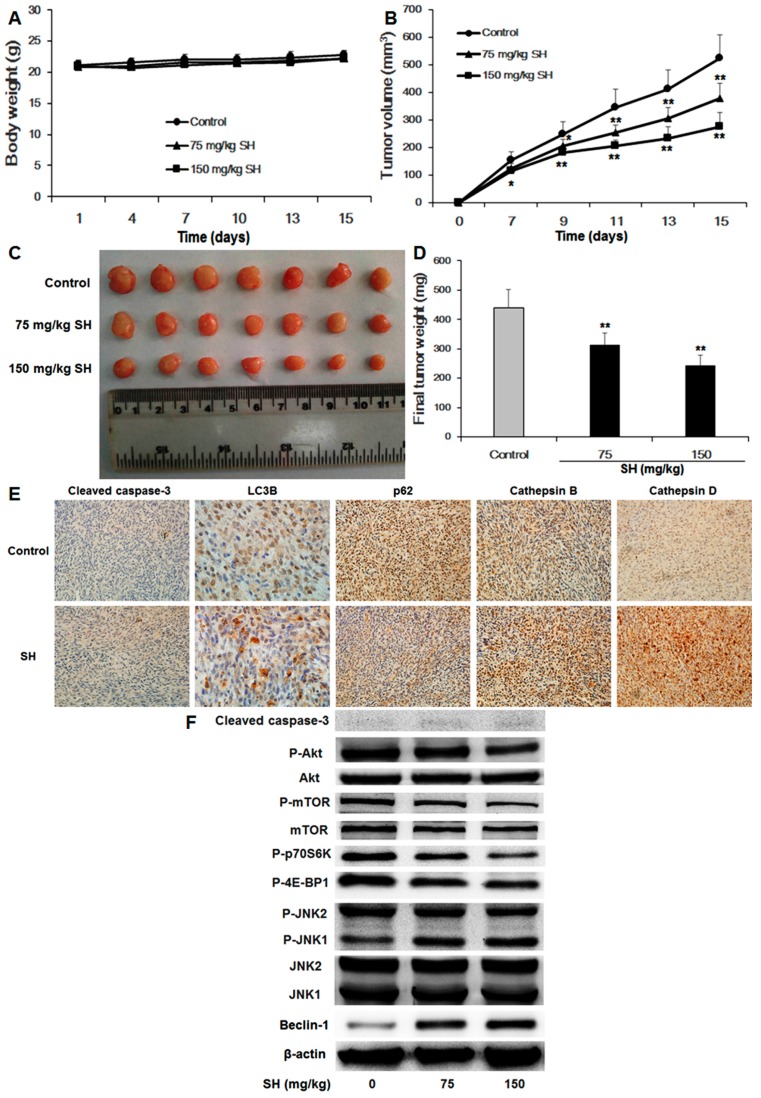
SH suppresses the growth of human glioblastoma tumors in vivo. (**A**) Body weight and (**B**) tumor volume were recorded every 2–3 days; (**C**) Images of tumors are shown; (**D**) Tumor weight was assessed on the fifteenth day after U87 cell implantation. (**E**) Immunohistochemical staining of cleaved caspase-3, LC3B, p62, cathepsin B and cathepsin D in tumor sections treated with physiological saline or SH (150 mg/kg). The panels are representative overview images taken at 400× magnification, with the exception of the immunohistochemical staining of LC3B (1000× magnification); (**F**) The indicated proteins from transplanted mouse U87 human glioblastoma tissues were examined via Western blotting. β-actin was used as the loading control. Data are represented as the mean ± SEM of seven mice in each group. * *p* < 0.05, ** *p* < 0.01, SH-treated group compared with the control group not treated with SH.

Nude mice bearing U87 tumors were treated with SH or physiological saline for 14 days. After treatment with SH, no significant changes in body weight were observed in all groups (Figure 7A). Dose-dependent decreases in tumor volume (Figure 7B,C) and tumor weight (Figure 7D) were found in the 75 and 150 mg/kg SH-treated groups (*p* < 0.05 or *p* < 0.01, respectively). Consistent with the results of the in vitro experiments, immunohistochemical analysis demonstrated that SH treatment had no effect on cleaved caspase-3, an apoptosis marker; LC3B staining was diffusely distributed within the cytoplasm, little LC3B puncta staining was detected in the control group, whereas a great deal of LC3B puncta staining, indicative of autophagosomes, was observed in the tumor tissues treated with SH; moreover, SH treatment substantially enhanced the expression levels of cathepsin B and cathepsin D and downregulated the expression level of p62 in the tumor tissues, compared with the control group (Figure 7E). Subsequently, to clarify the mechanisms of autophagy induced by SH in vivo, we investigated various proteins in the tumor tissues of nude mice transplanted with U87 cells. Similar results were obtained, compared with the in vitro experiments (Figure 7F). These findings revealed that SH initiated the autophagy-lysosome pathway in both in vitro and in vivo experiments. Furthermore, during the treatments, no behavioral abnormalities were found, and no morphological abnormalities in major organs, such as the lungs, liver, pancreas and kidneys, were observed.

## 3. Discussion

For the first time, our study revealed that SH could suppress U87 and SF767 cell proliferation in vitro and in vivo. To clarify the mechanisms by which SH mediated U87 and SF767 cell death, we detected the effects of SH in ROS generation and partial activation of the autophagy-lysosome pathway.

Autophagy is an evolutionarily conserved catabolic process beginning with the formation of autophagosomes. Autophagosomes take part in the recycling of cellular components by sequestering misfolded proteins and damaged organelles and eliminating them through lysosomal degradation [41,42]. Depending on cellular conditions as well as the duration and strength of the stress stimuli, autophagy can inhibit or promote cancer cell death [43,44]. Nevertheless, the molecular mechanisms of these dual effects of autophagy remain unclear. Normally, autophagy facilitates a portion of the organelles and cytoplasm into autophagic vesicles as one of the survival responses to stress. Under persistent autophagic stimuli, overmuch autophagy exhausts critical proteins and organelles, ultimately resulting in caspase-independent cell death. In this regard, autophagy might be employed as a therapeutic target only if it can be strongly activated in cancer cells. Lu et al. found that SH caused apoptosis in human hepatocellular carcinoma cells via the caspase pathway [8]. However, we did not observe any increase in the expression of cleaved caspase-3 in either culture conditions or tumor tissues from nude mice during SH treatment. The caspase-3 inhibitor Ac-DEVD-CHO and the pancaspase inhibitor Z-VAD-FMK did not decrease the increase in U87 and SF767 cell death mediated by SH. In summary, these observations reveal that SH-mediated cell death does not occur through caspase-dependent apoptosis in U87 and SF767 cells. Instead, we discovered that SH can trigger autophagy, as demonstrated by the formation of autophagosomes, AVs stained with MDC and upregulation of the autophagy marker protein LC3B-II. In addition, blocking autophagy using Beclin-1 or ATG5 siRNA reduced the SH-mediated increase in the expression levels of LC3B-II, while CQ pretreatment markedly enhanced LC3B-II accumulation in the two cell lines treated with SH. These data demonstrate that SH enhances autophagic flux in the human glioblastoma cells. To verify the precise effect of altered autophagy in SH-mediated cell death, we investigated the role of RNA interference against Beclin-1 and ATG5 or an autophagy activator (Rapa) and autophagy inhibitors (3-MA, WORT and CQ) in SH-induced glioblastoma cell death. Interestingly, the results suggested that inhibition of autophagy augmented cell viability; conversely, activation of autophagy decreased cell viability in the two cell lines treated with SH. Thus, these findings provided powerful evidence indicating that the activation of autophagy leads to SH-mediated cell death in the human glioblastoma cells. Some anticancer agents, such as temozolomide and arsenic trioxide, have been shown to induce autophagy-mediated cancer cell death without triggering caspase-dependent apoptosis [44,45].

An increasing number of studies have demonstrated that the Akt-mTOR pathway controls autophagy induction, and anticancer agents can inhibit tumor survival and growth via suppression of the Akt-mTOR pathway along with stimulation of autophagy [46,47]. In addition to its effects in cancer cell proliferation, the mTOR pathway plays an essential role in cancer formation. Accumulating data demonstrate that anomalies in protein synthesis downstream of mTOR, at the expression level of 4E-BP1, play an important role in tumor formation [48]. Moreover, 4E-BP1 has been reported to be involved in the oncogenic roles of Akt signaling in tumor progression, cell growth and mRNA translation [49]. Furthermore, the mTOR pathway has been demonstrated to be a key modulator in glioblastoma cell proliferation and is implicated in cancer pathogenesis [50,51]. Here, we suggest that SH dose-dependently reduces the phosphorylation of p70S6K, 4E-BP1, Akt and mTOR, while enhancing the expression levels of autophagy marker proteins in vivo and in vitro. In contrast, pretreatment with the Akt activator IGF-1 not only partially reversed the suppression of the Akt-mTOR pathway induced by SH but also markedly inhibited the induction of autophagy by SH, as demonstrated by the augmented phosphorylation of p70S6K, 4E-BP1, Akt and mTOR and the reduction of the accumulation of LC3B-II and the expression levels of ATG5 in U87 and SF767 cells treated with SH. These findings suggest that suppression of the Akt-mTOR pathway is associated with triggering of autophagy mediated by SH, and these data are consistent with the strong inhibition of mTOR observed in the cells generating autophagy induction. In addition, previous studies have demonstrated that the JNK signaling pathway is involved in caspase-independent autophagy-mediated cell death, and activated JNK1-mediated phosphorylation of Bcl-2 enhances free Beclin-1 levels through discharging Beclin-1 from the Beclin-1-Bcl-2 complex, consequently promoting autophagy-induced cell death [29,30,31,52]. Consistent with these observations, SH-induced autophagy was illustrated to be partially attributed to the JNK pathway activation in vitro and in vivo.

ROS, which can be produced via numerous mechanisms, are non-radical molecules or highly reactive oxygen free radicals that are primarily generated from mitochondria and nicotinamide adenine dinucleotide phosphate oxidases [53,54]. ROS act as significant versatile signaling molecules that modulate various cellular pathways and therefore play central roles in cell fate determination [53,55,56]. At low levels, ROS are associated with many vital cellular signaling pathways. However, ROS can also cause cellular damage resulting in cell death, including autophagy-mediated cell death [57]. We therefore examined whether SH-induced autophagy and cell growth inhibition are also dependent on ROS production. We demonstrated that SH dose-dependently augmented ROS generation, which was accompanied by increased autophagy in both U87 and SF767 cells. In contrast, the suppression of ROS by pretreatment with the ROS scavenger NAC reduced not only the increase in the levels of intracellular ROS but also the autophagy induction and cell growth inhibition mediated by SH in the two cell lines. These data indicate that the anti-human glioblastoma effects of SH are at least partially dependent on ROS production. Taken together, these findings indicate that SH triggers ROS generation, which is necessary for SH-mediated autophagy and cell growth suppression in both human glioblastoma cell lines. We further determined that the SH-induced increase in the levels of ROS is upstream of the Akt-mTOR and JNK pathways. Previous reports have shown that an alkaloid extracted from lotus can induce autophagic cell death regulated by ROS production upstream of the Akt-mTOR pathway [46]. Moreover, Kamata et al. found that tumor necrosis factor-α-dependent ROS generation results in JNK activation and cell death [58]. Additionally, ROS-mediated activation of the JNK pathway plays a causal role in the enhancement of autophagic flux via upregulation of Atg7 and Atg5 [30,40]. Consistent with the above findings, SH-mediated autophagic cell death in both human glioblastoma cell lines used in the present study was demonstrated to occur through a similar process.

As autophagy proceeds inside the lysosomal compartment, at some point, lysosomal biogenesis determines the autophagic flux. TFEB is a master gene for lysosomal biogenesis and controls this process by manipulating the expression of lysosomal and autophagy genes [59]. Pharmacological suppression of mTOR can activate TFEB through driving its dephosphorylation at serine residues and thereby facilitating its nuclear translocation; after translocation into the nucleus, TFEB can regulate lysosomal biogenesis and autophagosome formation [33,60,61]. Consistent with these previous findings, we illustrated that SH may promote lysosomal biogenesis by triggering the nuclear translocation of TFEB via mTOR inhibition. However, the precise mechanisms involved still require further investigation.

Nowadays, SH is mainly and widely applied in the clinical treatment of rheumatoid diseases. However, the studies of anti-cancer effects of SH are in the basic research phase, to our knowledge, there are no related reports of clinical experiments on that of SH. Meanwhile, preclinical experiments need to be further improved and confirmed. Although there are no significant influence on the body weight of nude mice, no behavioral abnormalities and no morphological abnormalities in major organs observed in the present experiments in vivo, previous study has shown that the main adverse reactions of SH in the clinical treatment of rheumatoid diseases are itchy skin, rash, gastrointestinal reaction, mild decline in the number of leukocytes and moderate elevation in the level of aminotransferase, as well as they can be quickly recovered by symptomatic treatments [62]. With consideration of them, the dosage of SH could be decreased in the potential clinical applications in many ways to reduce its adverse reactions, such as by modifying its chemical structure, using different administration methods and generating sustained-release dosage forms, although these need to be proceeded in further studies.

**Figure 8:**
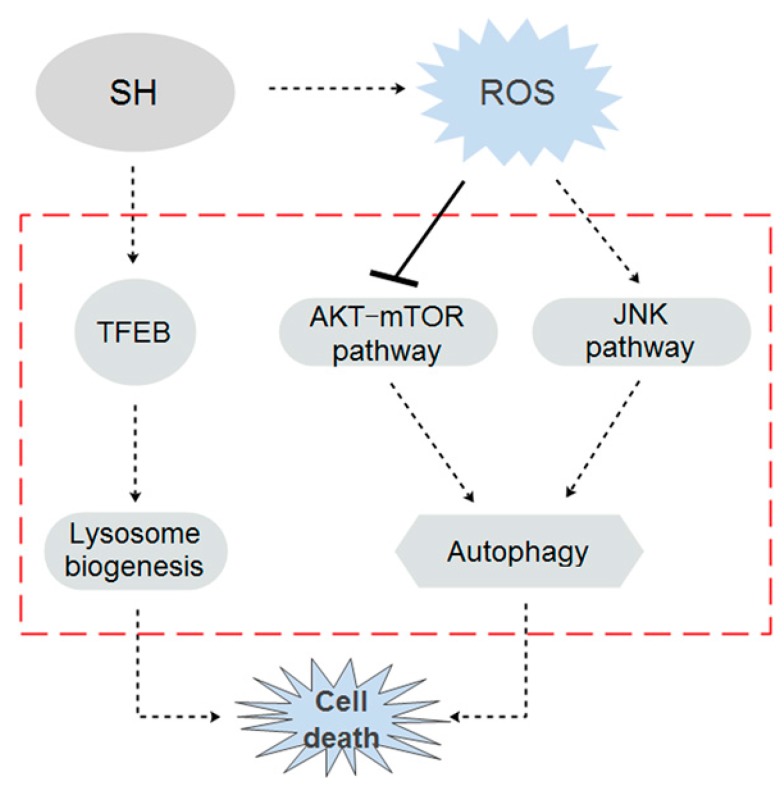
Proposed model delineating the mechanisms of SH action in the human glioblastoma cells.

In conclusion, this is the first time, to our knowledge, that the in vitro and in vivo anti-proliferative effects of SH on human glioblastoma have been observed, and a novel mechanism of action of SH on cancer cells in autophagy induction has been revealed (Figure 8). The major findings include the following: (1) SH treatment caused autophagy, but not apoptosis, in U87 and SF767 cells; (2) SH induced autophagy through the ROS-, Akt/mTOR- and JNK-dependent pathway; (3) SH may facilitate lysosome biogenesis by activating TFEB; and (4) SH inhibited the growth of human glioblastoma xenografts, without showing significant toxicity, when administered systemically. These findings provide a theoretical basis for the further development of clinical applications of SH in treating human glioblastoma.

## 4. Materials and Methods

### 4.1. Reagents and Antibodies

SH was purchased from Zhengqing Pharmaceutical Group (Huaihua, China). NAC, Rapa, CQ, 3-MA, SP600125, MDC and AO were purchased from Sigma-Aldrich (St. Louis, MO, USA). IGF-1 was purchased from Peprotech (Rocky Hill, NJ, USA). Probes DCFH-DA, LTR, WORT, Z-VAD-FMK and Ac-DEVD-CHO were purchased from Beyotime (Shanghai, China). Annexin V-FITC Apoptosis Detection kit was purchased from BD Biosciences (Franklin Lakes, NJ, USA). CCK-8 was purchased from Dojindo Laboratories (Kumamoto, Japan). Lipofectamine^TM^ 2000 transfection reagent was purchased from Invitrogen (Carlsbad, CA, USA). Antibodies against p62/SQSTM1, mTOR, phospho-mTOR (ser2448), Akt, phospho-Akt (ser473), JNK, phospho-JNK (thr183 and tyr185), ATG5, Beclin-1, LAMP-1, cathepsin D, phospho-Bcl-2 (ser87), and β-actin were obtained from Santa Cruz Biotechnology (Santa Cruz, CA, USA). Antibodies against Histone H3, phospho-p70S6K (thr389), phospho-4E-BP1 (ser65), cleaved caspase-3, and TFEB were obtained from Cell Signaling Technology (Danvers, MA, USA). Antibodies against LC3B and cathepsin B were obtained from Abcam (Cambridge, UK). Secondary antibodies included anti-mouse IgG-horseradish peroxidase (HRP), anti-rabbit IgG-HRP and anti-goat IgG-HRP, which were obtained from Santa Cruz Biotechnology (Santa Cruz, CA, USA).

### 4.2. Cell Culture and Treatment

Human glioblastoma cell lines U87 and SF767 were purchased from the cell resource center of the Chinese Academy of Medical Science (Beijing, China). U87 cells were maintained in minimum essential medium/Earle’s balanced salt solution (MEM/EBSS) supplemented with 1% non-essential amino acids, penicillin (100 units/mL), streptomycin (100 μg/mL), and 10% fetal bovine serum (FBS) in a humidified atmosphere of 5% CO_2_ and 95% air at 37 °C. SF767 cells were maintained in Dulbecco’s modified Eagle’s medium/HIGH glucose culture medium supplemented with penicillin (100 unit/mL), streptomycin (100 μg/mL), and 10% FBS in a humidified atmosphere of 5% CO_2_ and 95% air at 37 °C. Cells in mid-logarithmic growth were used for the following experiments. When they reached 75% confluence, cells were treated with the indicated concentrations of SH. Where indicated, NAC (5 mM), 3-MA (5 mM), WORT (10 μM), CQ (20 μM), Rapa (100 nM), IGF-1 (100 ng/mL), SP600125 (10 µM), Z-VAD-FMK (50 µM) and Ac-DEVD-CHO (50 µM) were added 1 h before SH administration.

### 4.3. Cell Viability Measurement

The CCK-8 assay was used to measure cell viability. According to the manufacturer’s protocol, cells were cultured in a 96-well plate and exposed to various treatments, as indicated, for 24, 48 or 72 h. Subsequently, 10 µL of CCK-8 was added to each well, and the plates were incubated at 37 °C for 1 h. Optical density values were determined at 450 nm with an Infinite^TM^ M200 Microplate Reader (Tecan, Männedorf, Switzerland). Cell viability was expressed as the percentage of control. Each experiment was performed in triplicate.

### 4.4. Lactate Dehydrogenase (LDH) Assay

The cytotoxicity was assessed by measurement of LDH release from the damaged cells using LDH Kit (Roche, Basel, Switzerland) according to the manufacturer’s directions.

### 4.5. Colony Formation Assay

Human glioblastoma cells were seeded into wells of a 6-well plate with 500 cells per well for 24 h. The cells were treated with or without the indicated concentrations of SH, and the culture medium containing SH was removed after 24 h. Subsequently, the cells were washed with phosphate-buffered saline (PBS) and cultured for another 10 days to allow colony formation. The culture medium was changed every 3 days. Then, the colonies were washed with PBS, fixed using 4% paraformaldehyde and stained with 0.5% crystal violet. The number of macroscopically observable colonies was counted and compared with the control. All experiments were performed in triplicate. 

### 4.6. Annexin V-FITC/Propidium Iodide (PI) Assay

Annexin V-FITC/PI staining was used to assess the effect of SH on apoptosis with an Annexin V-FITC Apoptosis Detection kit, following the manufacturer’s directions. Briefly, cells were cultured for 24 h in 6-well plates and then exposed to various treatments, as indicated, for 48 h. After washes with PBS, the cells were detached in trypsin and centrifuged (5 min, 4 °C, 1000 rpm), and 1 × 10^5^ cells were resuspended in 100 μL of 1× binding buffer. Subsequently, the cells were incubated with PI (5 μL) and Annexin V-FITC (5 μL) for 15 min at room temperature (25 °C) in the dark. Finally, 400 μL of 1 × binding buffer was added, and the samples were measured for apoptosis by a FACScan flow cytometer (Becton Dickinson, Franklin Lakes, NJ, USA). 

### 4.7. ROS Assay

For measurement of ROS levels, the cells were cultured in a 96-well plate. When the cells reached 75% confluence, they were exposed to the indicated treatments for 24 h. After washes with PBS, the cells were incubated with DCFH-DA (10 μM) at 37 °C for 20 min in the dark. At the end of the incubation period, the cells were washed again with PBS three times. Subsequently, relative fluorescence was analyzed with an Infinite^TM^ M200 Microplate Reader at excitation and emission wavelengths of 488 and 525 nm, respectively, three times. Cellular fluorescence intensity was expressed as a percentage of the control.

### 4.8. Transmission Electron Microscopy

U87 and SF767 cells were treated with SH (0.5 mM) for 24 h. Thereafter, the cells were harvested and fixed in a solution containing 0.1% glutaraldehyde and 2% paraformaldehyde in 0.1 M sodium cacodylate for 2 h, and postfixed with 1% O_S_O_4_ for 1.5 h, then washed and stained in 3% aqueous uranyl acetate for 1 h. Subsequently, the samples were washed, dehydrated through a graded alcohol series and embedded in Epon-Araldite resin. Finally, Ultra-thin sections were cut on a Reichert ultramicrotome, counter-stained with 0.3% lead citrate and observed using a Philips EM420 electron microscope (Philips, Eindhoven, The Netherlands).

### 4.9. Confocal Microscopy

Formation of acidic vesicular organelles, a morphological characteristic of autophagy, was examined by AO staining. When the cells reached 75% confluence, they were exposed to the indicated treatments for 24 h. After the cells were washed with PBS, they were stained with 1 μg/mL AO in PBS at 37 °C for 20 min in the dark. At the end of incubation, the cells were washed with PBS three times and imaged by a laser scanning confocal microscopy (Green fluorescence was acquired with excitation wavelength 488 nm, emission wavelength 520/560 nm; red fluorescence was acquired at excitation wavelength 488 nm, emission wavelength 575/640 nm).

The autofluorescent agent MDC was applied as a specific autophagolysosome marker to determine the autophagic process. When the cells reached 75% confluence, they were exposed to the indicated treatments for 24 h. Autophagic vacuoles were labeled with MDC by incubating the cells with 50 μM MDC for 15 min, then the cells were analyzed under a laser scanning confocal microscopy (excitation, 390 nm; emission, 460 nm).

Laser scanning confocal microscopy of Human glioblastoma cells loaded with DCFH-DA. The DCFH-DA probe was used to measure the levels of ROS. In brief, according to the manufacturer’s protocol, when the cells reached 75% confluence, they were exposed to the indicated treatments for 48 h. The cells were incubated with DCFH-DA (10 μM) in PBS for 20 min, and the samples were observed via laser scanning confocal microscopy (excitation, 488 nm; emission, 525 nm).

Laser scanning confocal microscopy of human glioblastoma cells loaded with LTR. When the cells reached 75% confluence, they were exposed to the indicated treatments for 24 h. The cells were loaded with red-fluorescing LTR (75 nM) in cell culture medium for 2 h, then the samples were measured by a laser scanning confocal microscopy (excitation, 577 nm; emission, 590 nm).

### 4.10. Small Interfering RNA (siRNA) Assay

SiRNAs for ATG5 (human) and Beclin-1 (human) were obtained from Santa Cruz Biotechnology, along with control siRNA. According to the manufacturer’s directions, the cells were transfected with 100 nM siRNA, and the medium containing Lipofectamine^TM^ 2000 was exchanged to fresh cell culture medium after 24 h. Subsequently, the cells were treated with the indicated treatments and used for the further experiments. 

### 4.11. Western Blot

The cells were lysed on ice in 1 × Lysis Buffer (Life Technologies, Carlsbad, CA, USA) with phosphatase inhibitor (Beyotime, Nantong, China) and complete protease inhibitor mixture (Roche Applied Science, Indianapolis, IN, USA). Nuclear and cytosolic fractions of the cells were isolated using nuclear and cytoplasmic protein extraction kits (Beyotime, Nantong, China) according to the manufacturer’s directions. Protein concentration was determined using the BCA Protein Assay Kit (Beyotime, Nantong, China). Approximately thirty to seventy micrograms of protein was separated using sodium dodecyl sulfate polyacrylamide gel electrophoresis (SDS-PAGE) and transferred to polyvinylidene fluoride (PVDF) membranes. The membranes were blocked in 5% skim dry milk (1.5 h) and incubated with primary antibodies overnight at 4 °C, followed by HRP-conjugated secondary antibodies for 1 h at room temperature. After the membranes were washed three times with Tris buffered saline-Tween, the bands were detected via enhanced chemiluminescence.

### 4.12. Animals and Treatments

All animal procedures were performed according to the protocol approved by the Animal Ethics Committee, China Academy of Chinese Medical Sciences, Beijing, China (approval number: 2017022). Five-week-old male BALB/c nude mice with body weights of approximately 20 g were obtained from the Beijing Vital River Laboratory Animal Technology Co., Ltd. (Beijing, China) and housed in specific pathogen-free barrier system of Animal Center, China Academy of Chinese Medical Sciences. After 1 week of acclimation, the nude mice were subcutaneously injected at the right flanks with U87 cells (1.5 × 10^6^) resuspended in 0.1 mL of media (1:1 Matrigel and PBS). Twenty-four hours after U87 cell implantation, the mice were randomly assigned to three groups of seven animals each. Animals in the control group were intraperitoneally (i.p.) injected with 0.1 mL of physiological saline per 10 g body weight once per day, and the treatment groups were injected i.p. with SH dissolved in physiological saline at a dose of 75 or 150 mg/kg of their body weight once per day for 14 days consecutively. Tumor sizes were measured every 2 days from the seventh day after U87 cell inoculation using calipers, and tumor volumes were calculated according to the standard formula: (width^2^ × length)/2 and expressed as mm^3^. Finally, the mice were sacrificed, and the tumor tissues were removed, measured, weighted, photographed and snap-frozen in liquid nitrogen for Western blot analysis or were fixed in 4% paraformaldehyde for immunohistochemical analysis, on the fifteenth day after U87 cell inoculation.

### 4.13. Immunohistochemistry

In brief, the tumor tissues were fixed in 4% paraformaldehyde at 4 °C for 72 h. Then, the selected samples were embedded in paraffin, sectioned and stained with hematoxylin (Sinopharm Chemical Reagent Co., Ltd., Beijing, China) and with antibody for cleaved caspase-3, LC3B, p62, cathepsin B or cathepsin D. The primary antibodies were used at the following dilutions: 1:200 for cleaved caspase-3, 1:400 for LC3B, 1:100 for p62, 1:100 for cathepsin B and 1:200 for cathepsin D. Finally, the sections were mounted with DPX mountant (Sigma, St. Louis, MO, USA), and the images were captured using microscopy (Leica, Wetzlar, Germany).

### 4.14. Statistical Analysis

All experimental data are expressed as the mean ± SEM, and all experiments were performed at least three times. The statistical analysis was performed by *t*-test and one-way analysis of variance, using SPSS 13.0 (SPSS Inc., Chicago, IL, USA). *p* < 0.05 (*) or *p* < 0.01 (**) was considered as statistically significant, and Turkey-Kramer was used as a post hoc test if *p* < 0.05.

## Abbreviations

SH	Sinomenine hydrochloride
LC3B	Microtubule-associated protein light chain 3B
ROS	Reactive oxygen species
Akt	Protein kinase B
mTOR	Mammalian target of rapamycin
JNK	c-Jun NH2-terminal kinase
NAC	*N*-acetylcysteine
IGF-1	Insulin-like growth factor-1
TFEB	Transcription factor EB
Beclin-1	Autophagy-related 6
Rapa	Rapamycin
CQ	Chloroquine
3-MA	3-methyladenine
WORT	Wortmannin
LTR	Lyso-Tracker Red
MDC	Monodancylcadaverin
AO	Acridine orange
p62/SQSTM1	Sequestosome 1
ATG5	Autophagy-related 5
LAMP-1	Lysosomal-associated membrane protein-1
Bcl-2	B-cell lymphoma-2
p70S6K	Ribosomal protein S6 kinase
4E-BP1	Eukaryotic translation initiation factor 4E-binding protein 1
siRNA	Small interfering RNA
AVs	Autophagic vacuoles
